# IL-1β induces rod degeneration through the disruption of retinal glutamate homeostasis

**DOI:** 10.1186/s12974-019-1655-5

**Published:** 2020-01-03

**Authors:** Hugo Charles-Messance, Guillaume Blot, Aude Couturier, Lucile Vignaud, Sara Touhami, Fanny Beguier, Lourdes Siqueiros, Valérie Forster, Nour Barmo, Sébastien Augustin, Serge Picaud, José-Alain Sahel, Alvaro Rendon, Antje Grosche, Ramin Tadayoni, Florian Sennlaub, Xavier Guillonneau

**Affiliations:** 1 Sorbonne Université, INSERM, CNRS, Institut de la Vision, 17 rue Moreau, F-75012 Paris, France; 2 Department of Ophthalmology, Hôpital Lariboisière, Paris, France; 3 CHNO des Quinze-Vingts, DHU Sight Restore, INSERM-DGOS CIC 1423, 28 rue de Charenton, F-75012 Paris, France; 4 Department of Ophthalmology, The University of Pittsburgh School of Medicine, Pittsburgh, PA 15213 USA; 5 Department of Physiological Genomics, Ludwig-Maximilians-Universität München, Grosshaderner Str. 9, D-82152 Planegg-Martinsried, Germany

**Keywords:** Age-related macular degeneration, Monocyte, Macrophage, Photoreceptor, Glutamate

## Abstract

**Background:**

Age-related macular degeneration is characterized by the accumulation of subretinal macrophages and the degeneration of cones, but mainly of rods. We have previously shown that Mononuclear Phagocytes-derived IL-1β induces rod photoreceptor cell death during experimental subretinal inflammation and in retinal explants exposed to IL-1β but the mechanism is unknown.

**Methods:**

Retinal explants were culture in the presence of human monocytes or IL-1β and photoreceptor cell survival was analyzed by TUNEL labeling. Glutamate concentration and transcription levels of gene involved in the homeostasis of glutamate were analyzed in cell fractions of explant cultured or not in the presence of IL-1β. Glutamate receptor antagonists were evaluated for their ability to reduce photoreceptor cell death in the presence of IL1-β or monocytes.

**Results:**

We here show that IL-1β does not induce death in isolated photoreceptors, suggesting an indirect effect. We demonstrate that IL-1β leads to glutamate-induced rod photoreceptor cell death as it increases the extracellular glutamate concentrations in the retina through the inhibition of its conversion to glutamine in Müller cells, increased release from Müller cells, and diminished reuptake. The inhibition of non-NMDA receptors completely and efficiently prevented rod apoptosis in retinal explants cultured in the presence of IL-1β or, more importantly, in vivo, in a model of subretinal inflammation.

**Conclusions:**

Our study emphasizes the importance of inflammation in the deregulation of glutamate homeostasis and provides a comprehensive mechanism of action for IL-1β-induced rod degeneration.

## Introduction

Age-related macular degeneration (AMD) is the leading cause of irreversible blindness in the industrialized world [1]. Geographic atrophy (GA), a clinical form of late AMD, is characterized by a slowly expanding lesion of the retinal pigment epithelium (RPE) and photoreceptors (PRs) [2]. So far, there is no available treatment to stop or even slow the degeneration of PR and RPE cells, and spare vision in patients. GA mononuclear phagocytes (MPs)—a family of cells that include inflammatory monocytes (Mos), macrophages, and resident macrophages such as microglial cells (MCs) [3]—accumulate in the subretinal space in the atrophic region and in a transitional zone, just peripheral to the RPE-loss, where the number of rods already drops dramatically compared with the retina more distant from the lesion [4, 5]. We previously showed that subretinal MPs accumulating in aged- and light-challenged mice are in part derived from circulating inflammatory Mos [5, 6]. Genetic or pharmacological inhibition of inflammatory MP recruitment strongly inhibits PR degeneration [6–10] indicating that inflammatory cells may participate in observed PR degeneration in GA patients.

IL-1β is induced in acute and chronic degenerative diseases of the brain [11]. In the retina, we previously showed in light-challenged mice, a model of dry AMD, that subretinal MPs expressed IL-1β which induces rod death and cone outer segment loss [12, 13]. The biological effects of IL-1β are mediated by the type I IL-1β receptor (IL-1R1), while its type II decoy receptor and IL-1 receptor antagonist inhibits its effect. Interestingly, in the central nervous system, IL-1R1 is poorly expressed on neurons in comparison with glial cells [14], suggesting that IL-1β primarily alters glial cell functions and potentiates neuronal injury indirectly [15]. Similarly, IL-1R1 immunoreactivity has consistently been observed on retinal astrocytes and Müller cells [16] and its expression on PRs remains controversial, suggesting that IL-1β might induce PR death indirectly.

Diverse stimuli have been shown to affect PR survival, including loss of trophic factors [17], metabolic changes [18], oxidative stress [19], and glutamate excitotoxicity [20]. In physiological conditions, glutamate homeostasis is finely tuned by a family of excitatory amino acid transporters (EAATs), mostly EAAT1/GLAST. EAATs transport the extracellular glutamate into Müller cells to prevent excitotoxicity, a pathological process resulting from glutamate receptor over-stimulation. Indeed, retinal neurons—including rod and cone PRs—express glutamate ionotropic receptors such as AMPA and NMDA receptors [21–24], and glutamate excitotoxicity has been demonstrated to be a major threat for PR survival [20]. Interestingly, IL-1β has been shown to modify glutamate homeostasis by (i) altering glutamate uptake and glutamine synthesis by Müller cells in models of ischemic retinopathies [25] and (ii) by increasing the transcription and the activity of the cystine/glutamate antiporter system *x*_c_^−^ in the hypoxic–ischemic brain astrocytes leading to exacerbated glutamate release [26].

We hypothesized that IL-1β produced by subretinal MPs disrupts retinal glutamate homeostasis, leading to PR cell death. We here show that IL-1β does not induce direct PR cell death but disturbs retinal glutamate homeostasis and induces extracellular glutamate accumulation. We show that CNQX—the inhibitor of non-NMDA glutamate ionotropic receptors—completely inhibits IL-1β-induced rod PR cell death emphasizing the importance of this mechanism.

## Methods

### Mice

Breeding pairs of *Cx3cr1*^*GFP/GFP*^ were obtained from The Jackson Laboratories. C57Bl6/J male mice were obtained from JANVIER Lab. The mice were aged between 8 and 15 weeks and were kept in a specific pathogen-free environment in a 12-h/12-h light/dark (100 lux) cycle with no additional cover in the cage and with water and normal chow diet available ad libitum.

### Light challenge model

Two- to three-month-old *Cx3cr1*^*GFP/GFP*^ mice were adapted to darkness for 6 h and pupils were fully dilated with 1% Atropin (Novartis). Animals were then exposed to green LED light (4500 Lux, JP Vezon équipements) for 4 days. On day 5, animals were kept for 10 h in normal light condition and received intraperitoneal injections of vehicule or Talampanel (2 mg/kg in 0.5 % Tween 20, Sigma-Aldrich) [27] every 2 h until sacrifice (5 injections). For each eye, IBA1 MPs were counted on whole RPE/choroidal flatmounts and on the outer segment side of the retina. Photoreceptor degeneration was quantified on TUNEL-labeled retinal flatmounts.

### Retinal flatmount preparation and immunohistochemistry

Immunohistochemistry on retinal/choroidal flatmounts was conducted as previously described [12]. Briefly, mice were killed by CO_2_ asphyxiation and enucleated. The globes were fixed in 4% PFA for 30 min, then rinsed in 1x PBS (pH 7.3). Retinal and RPE/choroid tissues were dissected intact from the globe, flatmounted, and processed for immunohistochemistry using the polyclonal goat anti-IBA1 (ab5076, Abcam) and the secondary anti-goat antibody conjugated with Alexa Fluor 488 (Life Technologies). Flatmounts were stained with the nuclear marker Hoechst (1:1000). Flatmounts images were captured with a DM5500 microscope (Leica) and analyzed by MetaMorph software (MolecularDevices).

### Terminal deoxynucleotidyl transferase UTP end labeling

Terminal deoxynucleotidyl transferase UTP end labeling (TUNEL) staining was performed according to the manufacturer’s protocol (In Situ Cell Death Detection Kit, Roche Diagnostics). Briefly, retinal flatmount or retina was fixed in 4% PFA for 30 min and washed in 1x PBS (pH 7.3). Flatmounts were then incubated for 60 min at 37 °C with the reaction mixture (In situ Cell Death Detection Kit) and the reaction was stopped by washing with 1x PBS. Nuclei were stained with Hoechst (Sigma-Aldrich). Flatmount images were captured with a DM 6000 microscope (Leica) or an Olympus Confocal microscope.

### Retinal cell sorting

Retinal cells were sorted according to a previously published protocol [28]. Isolated retina was incubated with papain (0.2 mg/ml, Roche, Mannheim, Germany) in Ca^2+^ -/Mg^2+^ -free phosphate-buffered saline containing 11 mM glucose, pH 7.4, for 30 min at 37 °C, followed by several washing steps with saline. After short incubation in saline supplemented with DNase I (200 U/ml), the tissue was triturated in extracellular solution (ECS, that contained (mM) 135 NaCl, 3 KCl, 2 CaCl_2_, 1 MgCl_2_, 1 Na_2_HPO_4_, 10 HEPES, and 11 glucose, adjusted to pH 7.4 with Tris) to obtain isolated retinal cells. After centrifugation, the supernatant was removed and the cells were resuspended and incubated in ECS containing biotinylated hamster anti-CD29 (clone Ha2/5, BD Biosciences, Heidelberg, Germany) for 15 min at 4 °C. After washes in ECS and centrifugation, cells were taken up in ECS containing anti-biotin MicroBeads (Miltenyi Biotec, Bergisch Gladbach, Germany) and incubated for 10 min at 4 °C. After an additional washing step in ECS, cell populations were separated using MACS® cell separation large cell columns (Miltenyi Biotec) according to the manufacturer’s recommendation. If microglia cells were isolated in addition to Müller cells, the retinal suspension was incubated with CD11b-microbeads (Miltenyi Biotec) for 15 min at 4 °C and positively selected using MACS® cell separation large cell columns (Miltenyi Biotec) before Müller cells were surface-labeled for MACS sorting.

### Mouse photoreceptor isolation

The protocol for retinal dissociation of the mouse retina was adapted from a previously published protocol [29]. The mouse retina was isolated and cut in small fragments in CO_2_-independent medium (Gibco). The retinal fragments were then washed twice in Ca^2+^-free Ringer’s solution and supplemented with 0.1 mM EDTA. They were then incubated with 0.2% activated papain (Worthington, Freehold, NJ, USA) in the same buffer for 20 min at 37 °C. Digestion was stopped by the addition of the same volume of neurobasal-A medium (Gibco) containing 2% FCS (Gibco), 0.1 mg/ml DNAse-1 (Sigma), and 1 mg/ml bovine-serum albumin. The retinal tissue was dissociated by repeated, gentle agitation. Cell supernatants were collected, pooled, and centrifuged at 800 rpm for 5 min. Cells were resuspended in a neurobasal-A medium containing 2% B27 supplement (Gibco), immediately plated and kept in the incubator (5% CO_2_/95% air) at 37 °C.

### Glutamic acid assay

Glutamate concentration was assessed in the supernatant of retinal explants cultured for 18 h with or without IL-1β (50 ng/ml), or in Müller cell lysate obtained after cell sorting using Amplex^TM^ Red Glutamic Acid Assay (MyQubit, Invitrogen). Reactions were conducted according to the manufacturer’s instructions.

### Monocytes preparations, monocyte-retinal co-culture, and retinal explants

PBMCs were isolated from heparinized venous blood from healthy volunteer individuals. Peripheral blood mononuclear cells (PBMCs) were isolated from blood by 1-step centrifugation on a Ficoll Paque layer (GE Healthcare) and sorted with the EasySep Human Monocyte Enrichment Cocktail (StemCells Technology, Grenoble, France). Human Mos (hMos) were seeded on polycarbonate filters floating on DMEM for 2 h. C57BL/6 J mouse retina was prepared and placed with the PRs facing 100,000 adherent hMos for 18 h at 37 °C. For TUNEL staining (In Situ Cell Death Detection Kit, Roche Diagnostics, Meylan, France), mouse retinal flatmounts were fixed in 4% PFA for 30 min, washed in 1x PBS (pH 7.3), and incubated for 90 min at 37 °C with the reaction mixture and the reaction was stopped by washing with 1x PBS. For immunohistochemistry, the flatmounts were incubated with Peanut agglutinin Alexa 488 (Thermofisher; 1:50) or IBA1 (Abcam; 1:400) overnight, and revealed with an appropriate antibody (Thermofisher). Nuclei were stained with Hoechst (Thermofischer; 1:1000). Flatmounts images were captured with an Olympus Confocal microscope.

### Quantification of cone outer segment volumes

Cone outer segment (CS) volumes were quantified using Imaris software (Bitplane) on stacks of confocal images, which contain the entire PR outer and inner segments, of PNA-stained retinal flatmounts. Four randomly chosen stacks (technical replicates) per retinal explant were taken. The software then calculated the volume of the PNA^+^ structures, which was subsequently divided by the number of PNA^+^ CS to yield the average individual CS volume in the stack. The CS volume of each retinal explant (biological replicate) was calculated as the mean of the 4 individual stacks.

### Quantifications of microglial cell density and morphology

Flatmount images of samples stained with IBA1 and Hoechst were captured with an Olympus Confocal microscope. Images were Z-projected using ImageJ 2.0 software. IBA1-positive cell perimeters (length around the periphery of each cell) and soma area (area of the cell body without any process) were manually measured on flatmount images using the ImageJ 2.0 software. For each retina, the microglial cell parameters were measured on a minimum of 4 representative images and a total number of 79 and 52 cells for control and IL-1β conditions respectively. Cell density was calculated by dividing the number of IBA1-positive cells by the surface of flatmount images.

### Western blot fragment analysis

Samples were diluted in 4X laemmli buffer and heated at 95 °C for 5–10 min before loading on the NuPAGE 4-12 % Bis-Tris gel. Each gel was loaded with PageRuler Prestained protein ladder (Thermo Fisher Scientific). Gels were transferred to nitrocellulose membranes (0.2 μm, GE Healthcare) by BioRad blotting system (Life Technologies) for Western Blotting according to the manufacturer’s protocol. Immunoblots were blocked with 5% skimmed milk powder in TBS 1X 0,2% Tween, then probed with the xCT antibody (Abcam ab175186) at a 1:500 dilution in the same buffer. Secondary antibodies, donkey anti-rabbit HRP (705-035-003, Jackson ImmunoResearch), were applied at a dilution of 1/5000 in blocking buffer. Chemoluminescence was performed using Pierce ECL-plus (32132, Thermo Fisher Scientific) and the ChemiDoc Imaging System (Biorad). Band intensity was quantified by ImageJ and normalized by β-actin.

### Reverse transcription and quantitative polymerase chain reaction

Reverse transcription quantitative polymerase chain reaction (RT-qPCR) was used to measure mRNA expression levels. Total RNA was extracted from cell fractions isolated from retinal explants, or total retinal explants, cultured for 18 h on polycarbonate filters floating on DMEM using the Nucleospin RNA (740955, Macherey-Nagel, Düren, Germany) according to the manufacturer’s instructions and converted to cDNA using oligo (dT) as primer and Superscript II (Life Technologies). Each RT assay was performed in a 20 μl reaction. Subsequent RT-qPCR was performed using cDNA, Sybr Green PCR Master Mix (Life Technologies), and the following sense and antisense primers (0.5 pmol/ml): *GluA-2* (5′-AAT GTG GAG CCA AGG ACT CG-3′ and 5′-CCA GCA TTG CCA AAC CAA GG-3′), *GluA-4* (5′-CGC CTA CTC TTG GCA ATG AC-3′ and 5′-GTC GAA CAG CGC AGA ACT C-3′), *GluK-1* (5′-TGC AGG ACT CGT CCT TTC TG-3′ and 5′-GTG AGA TTC CCA GCT CTT CC-3′), *Eaat1* (5′-TCA ATG CCC TGG GCC TAG TTG T-3′ and 5′-GGG TGG CAG AAC TTG AGG AGG-3′), *Eaat2* (5′-CCT CCA TCT GAG GAG GCC AAT-3′ and 5′-CAG CTG CCT AGC AAC CAC TTC T-3′), xCT (5′-AAA CTT GCT AAG CTC TGT GTT GG-3′ and 5′-CAC ATC ACA TGT TTG TAC ACT CG-3′), *Gs* (5′-CCC CAA CAA GCT GGT GCT AT-3′ and 5′-TCC ATT CCA AAC CAG GGG TG-3′), *Rps26* (5′-AAG TTT GTC ATT CGG AAC ATT-3′ and 5′-AGC TCT GAA TCG TGG TG-3′), β-actin (*Actb*) (5′-AAG GCC AAC CGT GAA AAG AT-3′ and 5′-GTG GTA CGA CCA GAG GCA TAC-3′), and *Gapdh* (GGT GAA GGT CGG TGT GAA CG and CTC GCT CCT GGA AGA TGG TG-3′). RT-qPCR was performed using the Applied Biosystems StepOne real-time PCR systems (Applied Biosystems) with the following profile: 10 min at 95 °C, followed by a total of 40 two-temperature cycles (15 s at 95 °C and 1 min at 60 °C). To verify the purity of the products, a melting curve was produced after each run according to the manufacturer’s instructions. Results were expressed as fold induction after normalization by the arithmetic mean of three housekeeping genes (HKG): *Rps26*, *Actb*, and *Gapdh*.

### Statistical analysis

GraphPad Prism 7 (GraphPad Software) was used for data analysis and graphic representation. All values are reported as mean ± SEM. Statistical analysis was performed by Kruskal–Wallis non-parametric tests or Mann–Whitney *U* tests for comparison among means depending on the experimental design. The *p* values are indicated in the figure legends.

## Results

### IL-1β induces rod photoreceptor degeneration indirectly

**Fig. 1:**
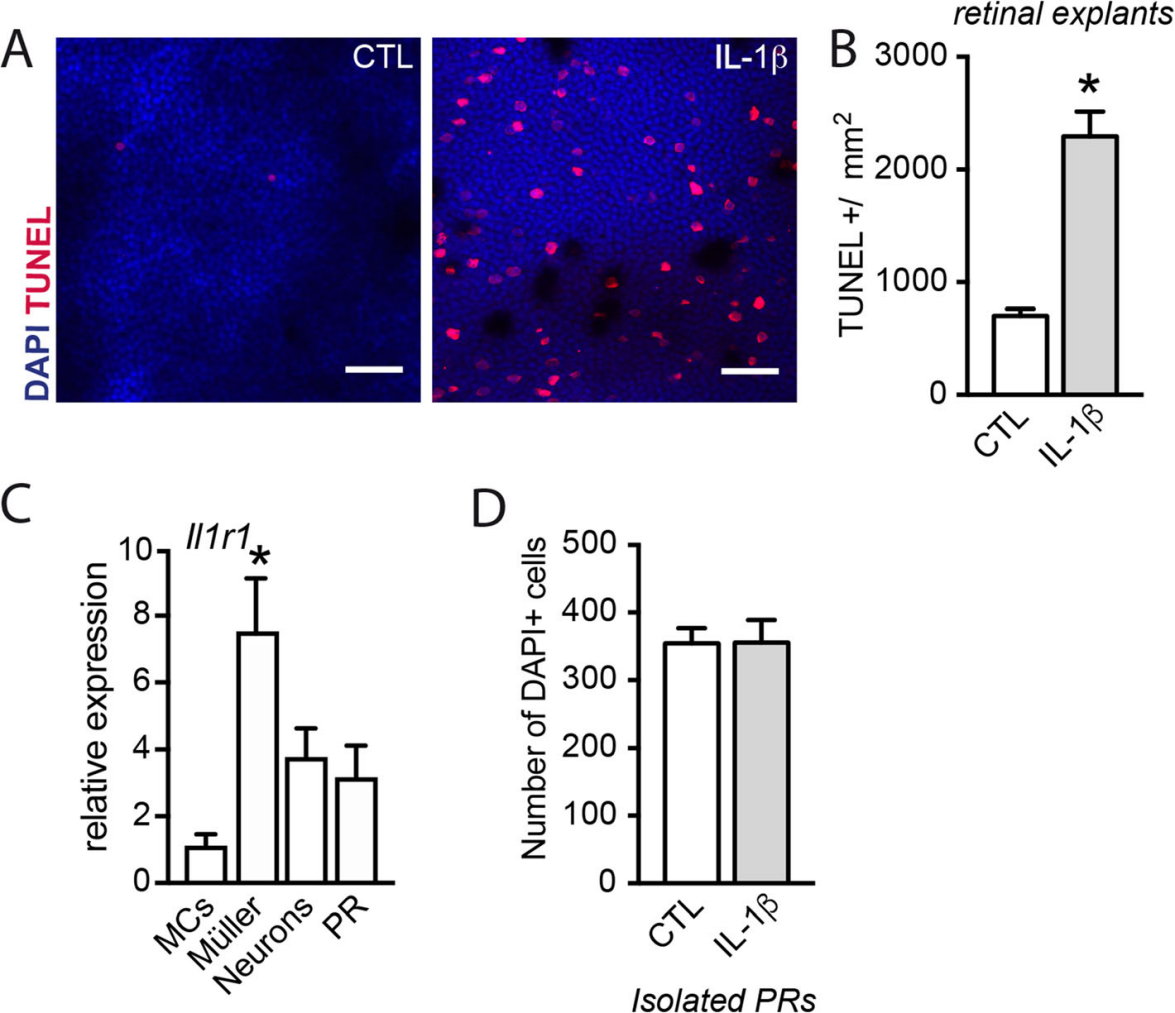
IL-1β induces rod photoreceptor degeneration indirectly. **a** Orthogonal projection of confocal Z stack images of the outer nuclear layer of TUNEL (red)- and Hoechst nuclear dye (blue)-stained mouse retinal explants after 18 h of culture with or without IL-1β (50 ng/ml). **b** Quantification of TUNEL^+^ nuclei in control and IL-1β (50 ng/ml) exposed mouse retinal explants (*n* = 10/group, Mann–Whitney test, *p < 0.0001). **c** RT-qPCR of *Il1r1* mRNA normalized with the mean of 3 HKG mRNAs in retinal cell fractions (MCs: CD11b^+^ cells; Müller cells: CD11b^−^CD31^−^CD29^+^ cells; neurons: CD11b^−^CD31^−^CD29^−^ and PR) isolated from total retina (*n* = 3/group, Kruskal–Wallis, Dunn’s post-test *p = 0.0023 versus MCs; *n* = 3/group. **d** Quantification of the number of DAPI^+^ isolated rod PRs cultured for 18 h with or without IL-1β (50 ng/ml) (*n* = 4/group)

We previously demonstrated that monocyte-derived IL-1β induces PR degeneration [12, 13]. Indeed, treatment of retinal explants with IL-1β (50 ng/ml) resulted in a 3.3-fold increase in the number of TUNEL-positive PR nuclei (Fig. 1a and b). However, RT-qPCR analysis of *Il1r1* mRNA of magnetic cell sorted retinal microglial cells (MCs), Müller cells, retinal neurons [28] and PR purified by differential centrifugation [29], confirmed previous reports of high *Il1r1* expression in Müller cells but low expression in PRs (Fig. 1c). Contrary to retinal explants, the addition of IL-1β (50 ng/ml) to cultured isolated PR did not result in detectable cell loss (Fig. 1d).

Together this result and the low expression of IL1R1 on PRs suggest that a non-PR cell population is needed to induce IL-1β-dependent rod degeneration.

### IL-1β increases extracellular glutamate concentration that can induce rod photoreceptor degeneration similar to IL-1β

**Fig. 2:**
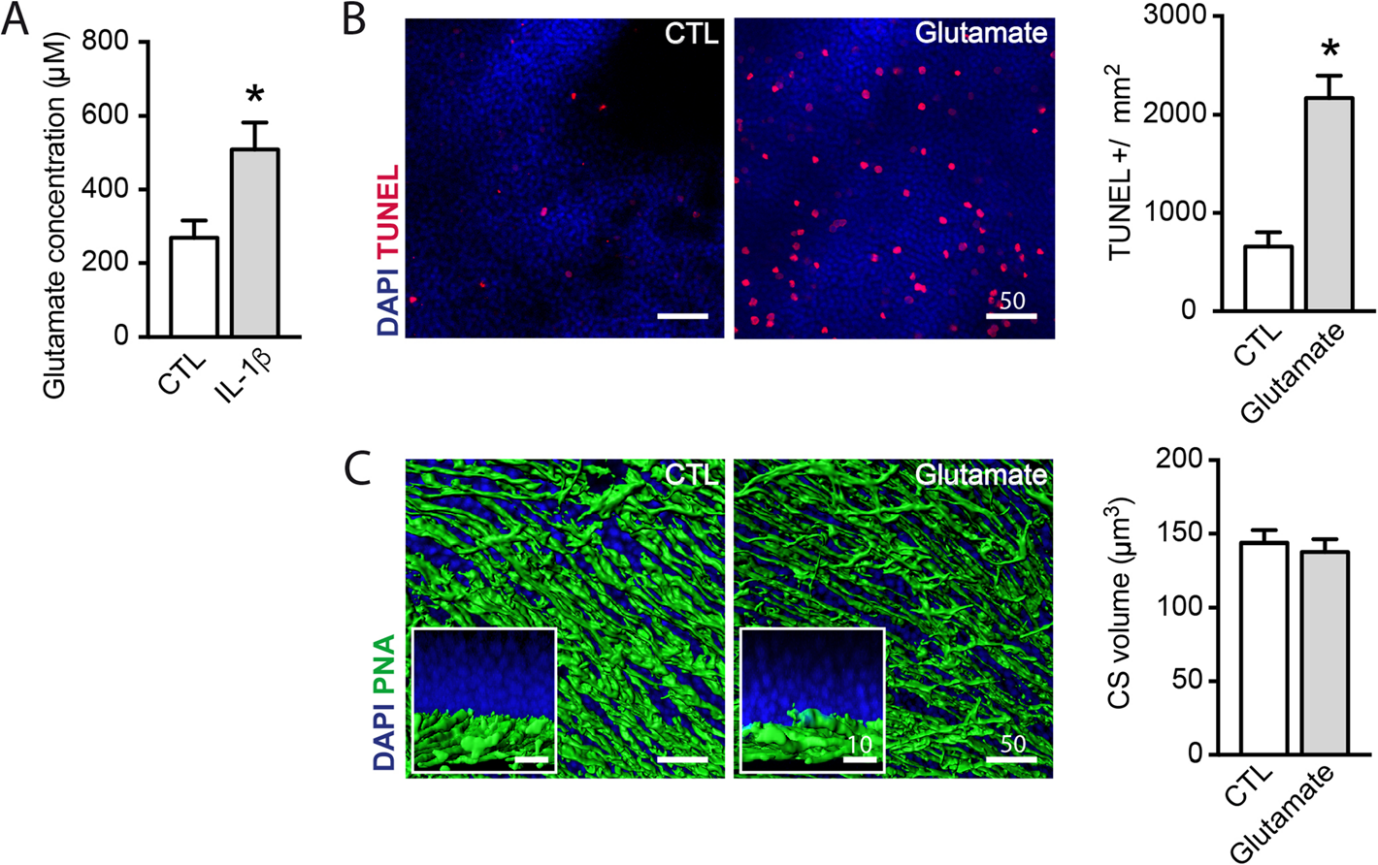
IL-1β increases extracellular glutamate concentration that can induce rod photoreceptor degeneration similar to IL-1β. **a** Glutamate concentration (μM) in the 18 h supernatant of mouse retinal explants cultured with or without IL-1β (50 ng/ml) (*n* = 8/group, Mann–Whitney test, *p = 0.0152). **b** Orthogonal projection of confocal Z stack images of the outer nuclear layer of TUNEL (red)- and Hoechst nuclear dye (blue)-stained mouse retinal explants after 18 h of culture with or without glutamate (1 mM), and quantification of TUNEL^+^ nuclei (*n* = 4/group, Mann–Whitney test, *p = 0.0286). **c** Oblique and perpendicular (Inset)—3D reconstructions of confocal Z stack images of the PR segments of mouse retinal explants after 18 h of culture with or without glutamate (1 mM), after peanut agglutinin (PNA, green) staining, and quantification of PNA^+^ outer segment volume (μm^3^) (*n* = 5/group, Mann–Whitney test)

IL-1β has been shown to disturb glutamate homeostasis [25, 30, 31] leading to indirect neuronal loss. Quantification of glutamate in the supernatants of retinal explants exposed or not to IL-1β (50 ng/ml) showed that IL-1β induced an increase in the extracellular content of glutamate from 270 to 508 μM (Fig. 2a). To evaluate whether an increase in extracellular glutamate could be responsible for the observed PR apoptosis, we next cultured retinal explants with 1 mM glutamate and analyzed cone and rod survival as previously described [12]. Similar to IL-1β, the addition of glutamate to the culture medium led to a 3.3-fold increase in TUNEL-positive PR nuclei (Fig. 2b). In contrast, glutamate did not induce cone loss or cone outer segment (CS) shortening (Fig. 2c), suggesting that glutamate mainly induces rod apoptosis. Together, our results demonstrate that IL-1β is sufficient to increase extracellular glutamate and that glutamate is sufficient to induce rod apoptosis.

### IL-1β disrupts glutamate homeostasis in Müller glial cells

Under physiological conditions, glutamate is released in the synapses and subsequently taken up by excitatory amino acid transporters (EAATs) of mainly Müller glial cells but also other cell types, preventing excitotoxic concentrations. In the outer retina, extracellular glutamate is mainly transported into Müller cells through EAAT1/GLAST, but EAAT2/GLT-1 on glial and non-glial cells also contributes to glutamate recapture [32]. After the uptake of extracellular glutamate by Müller cells, intracellular glutamate can be released from glial cells by a cystine/glutamate antiporter, the *x*_c_^−^ system, to import cystine to fuel glutathione synthesis necessary for anti-oxidant defense [33]. Alternatively, glutamate can be converted by the glutamine synthetase (GS) into glutamine to serve as source of energy or for protein or lipid synthesis.

**Fig. 3:**
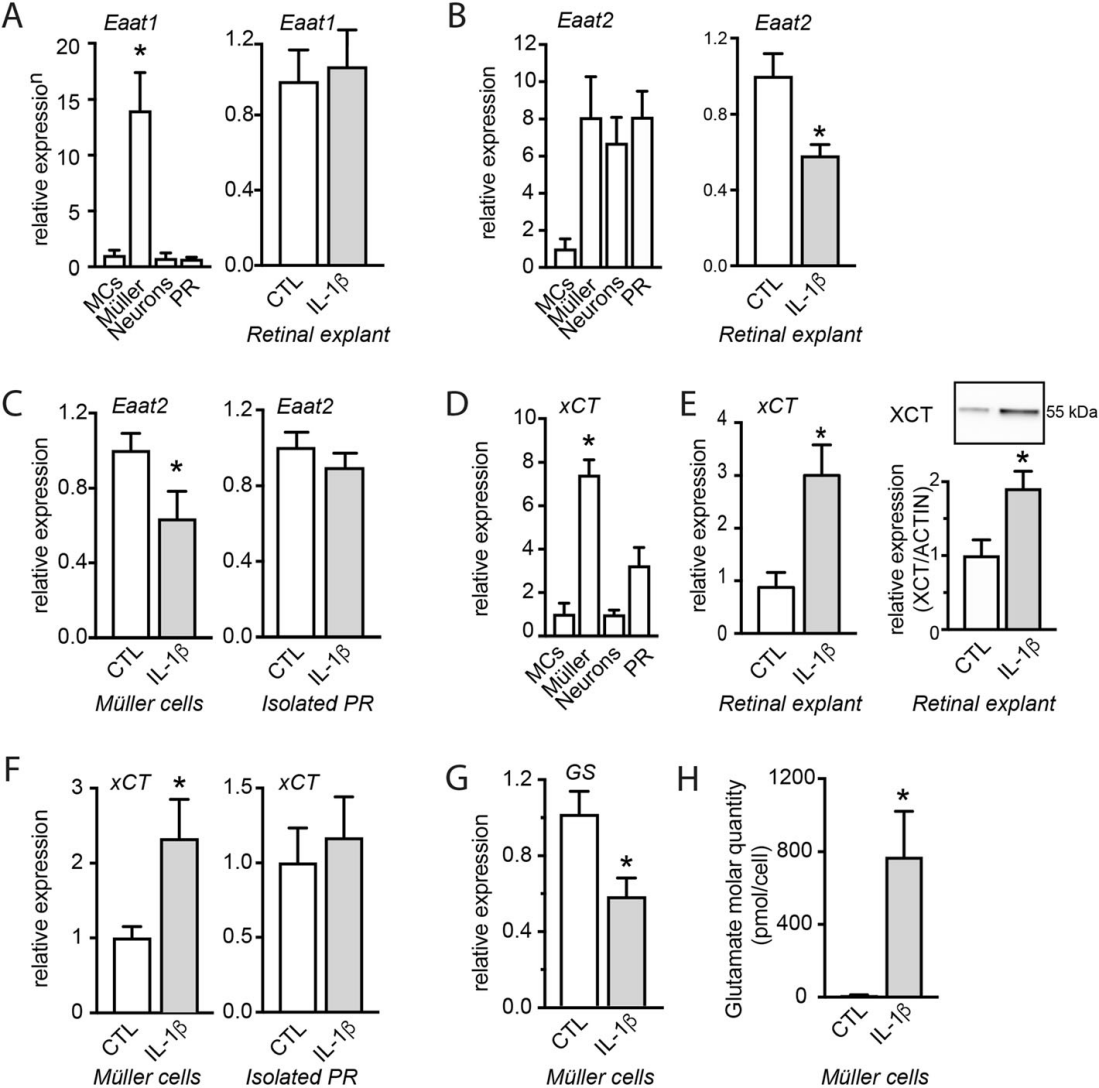
IL-1β disrupts glutamate homeostasis in Müller glial cells. **a** RT-qPCR of *Eaat1* mRNA normalized with the mean of 3 HKG mRNAs in (left) retinal cell fractions (MCs: CD11b^+^ cells; Müller cells: CD11b^−^CD31^−^CD29^+^ cells; neurons: CD11b^−^CD31^−^CD29^−^ and PRs) isolated from total retina (*n* = 3/group, Kruskal–Wallis, Dunn’s post-test *p = 0.0332 versus MCs; *n* = 3/group, Kruskal–Wallis, Dunn’s post-test) and in (right) total retinal explants cultured 18 h with or without IL-1β (50 ng/ml) (*n* = 5/group). **b** RT-qPCR of *Eaat2* mRNA normalized with the mean of 3 HKG mRNAs in retinal cell fractions isolated from (left) total retina and in (right) total retinal explants cultured 18 h with or without IL-1β (50 ng/ml) (*n* = 5/group, Mann–Whitney test; *n* = 5/group, Mann–Whitney test, *p = 0.0183). **c** RT-qPCR of *Eaat2* mRNA normalized with the mean of 3 HKG mRNAs in (left) Müller cell fraction (*n* = 6/group, Mann–Whitney test, *p = 0.0360) and (right) PRs isolated from retinal explants cultured with or without IL-1β (50 ng/ml) for 18 h (*n* = 6/group). **d** RT-qPCR of *xCT* mRNA normalized with the mean of *3 HKG* mRNAs in retinal cell fractions isolated from total retina (*n* = 4/group, Kruskal–Wallis, Dunn’s post-test *p = 0.0128 versus MCs). **e** (left) RT-qPCR of *xCT* mRNA normalized with the mean of 3 HKG mRNAs in retinal explants cultured with or without IL-1β (50 ng/ml) for 18 h (*n* = 6/group, Mann–Whitney test, *p = 0.0089). (right) Western blot analysis of XCT in retinal explants cultured with or without IL-1β (50 ng/ml) for 18 h (*n* = 5/group, Mann–Whitney test, *p = 0.0159). **f** RT-qPCR of *xCT* mRNA normalized with the mean of 3 HKG mRNAs in (left) Müller cell (*n* = 6/group, Mann–Whitney test, **p = 0.0303) and (right) PR fractions (*n* = 6/group) isolated from retinal explants cultured 18 h with or without IL-1β (50 ng/ml). **g** RT-qPCR of *Gs* mRNA normalized with the mean of 3 HKG mRNAs in Müller cell fraction isolated from retinal explants cultured with or without IL-1β (50 ng/ml) for 18 h (*n* = 6/group, Mann–Whitney test, *p = 0.0152). **h** Intracellular glutamate molar quantity (pmol/cell) measured in the lysate of the Müller cell fraction isolated from retinal explants cultured 18 h with or without IL-1β (50 ng/ml) (*n* = 5/group, Mann–Whitney test, *p = 0.0152)

To investigate how IL-1β leads to the accumulation of extracellular glutamate (Fig. 2a) we first analyzed the expression of *Eaat*s. RT-qPCRs of *Eaat1* mRNA of magnetic cell sorted retinal microglial cells, Müller cells, retinal neurons additionally to PRs purified by differential centrifugation, confirmed that *Eaat1* mRNA was almost exclusively expressed by the CD11b^−^ CD31^−^ CD29^+^ Müller cell fraction but not regulated by IL-1β (Fig. 3a). *Eaat2* was expressed by Müller cells, neurons (CD11b^−^ CD31^−^ CD29^−^ fraction) and PRs and IL-1β induced a 1.7-fold downregulation of *Eaat2* mRNA expression in retinal explants (Fig. 3b). RT-qPCRs of *Eaat2* mRNA of magnetic cell sorted retinal cells revealed that *Eaat2* mRNA was downregulated in Müller cells but not in PRs (Fig. 3c).

Next, RT-qPCR performed on isolated cell fractions confirmed that xCT mRNA, a subunit of *x*_c_^-^, is highly expressed in Müller cells, and to a lesser extent in PRs, compared with the CD11b^+^ MCs fraction (Fig. 3d). IL-1β (50 ng/ml) exposure significantly upregulated *xCT* mRNA expression and XCT protein in retinal explants (Fig. 3e). *xCT* mRNA expression was mainly upregulated in Müller cells, while the expression in isolated PRs remained stable (Fig. 3f), confirming previous reports [34, 35].

Similarly, *Gs* mRNA expression was decreased 1.7-fold in Müller cells (Fig. 3g) after addition of IL-1β, indicating a putative impairment of glutamate conversion into glutamine. Indeed, the intracellular glutamate content of Müller cells sorted from IL-1β-exposed retinal explants contained an average of 770 pmol/cell, while it was barely detectable in Müller cells from control explants (Fig. 3h).

Taken together, our data show that IL-1β impairs the conversion of glutamate into glutamine in Müller cells, leading to increased intracellular glutamate concentrations. IL-1β-induced increased XCT likely increases glutamate export from Müller cells. This deregulation is likely responsible for the increase of extracellular glutamate induced by the exposure to IL-1β (Fig. 2a).

### Microglial cells do not participate directly to glutamate homeostasis disruption induced by IL-1β

**Fig. 4:**
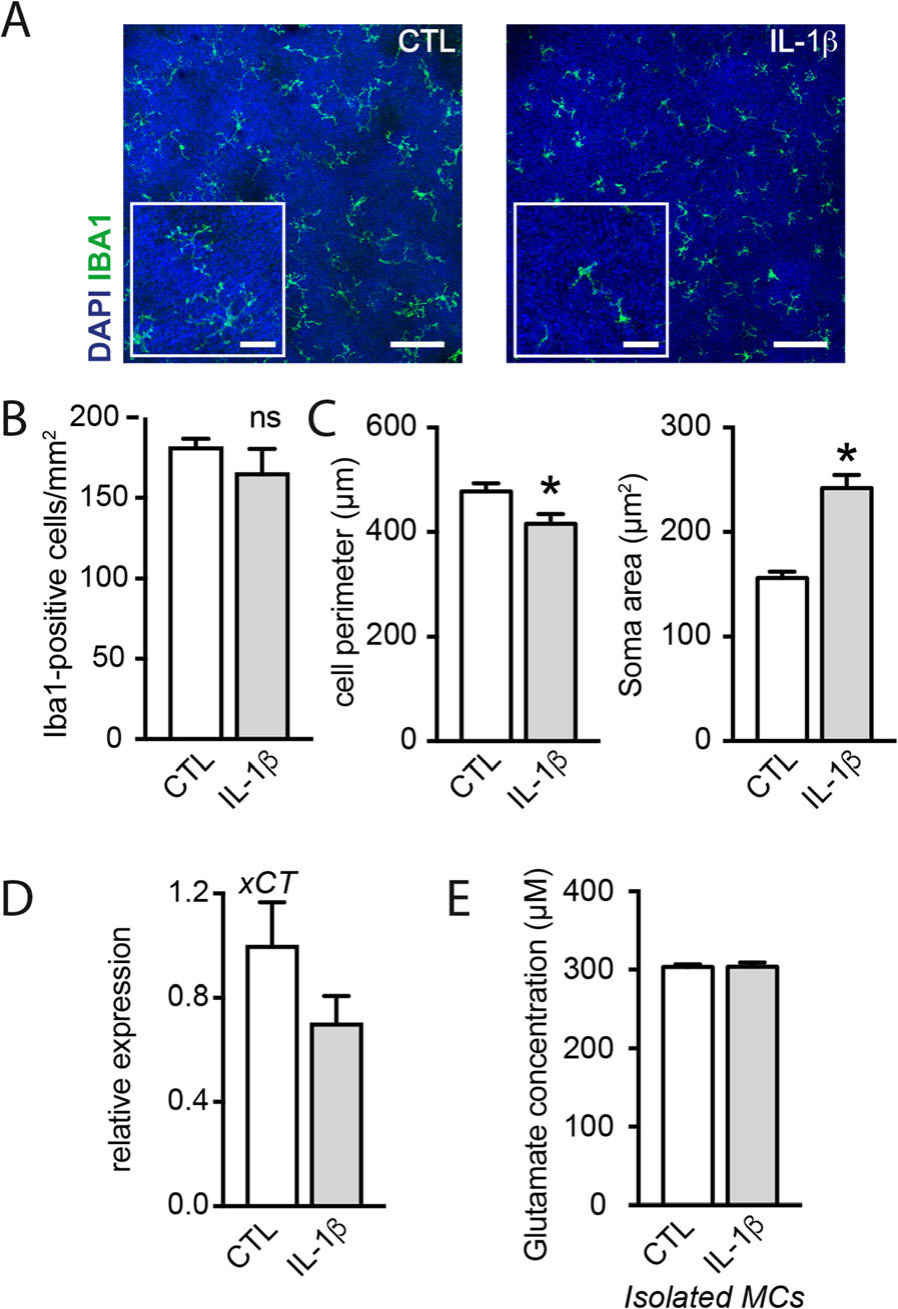
Microglial cells do not participate directly to glutamate homeostasis disruption induced by IL-1β. **a** Orthogonal projection of confocal Z stack images of the outer nuclear layer of IBA1 (green)- and Hoechst nuclear dye (blue)-stained mouse retinal explants after 18 h of culture with or without IL-1β (50 ng/ml). **b** Number of IBA1-postive cells per mm^2^ in the outer nuclear layer of mouse retinal explants after 18 h of culture with or without IL-1β (50 ng/ml) (*n* = 4 per group). **c** Quantification of the cell perimeter and the soma area of microglia from retinal explants cultured 18 h with or without IL-1β (50 ng/ml) (*t* test, *p = 0.0133 and *p < 0.0001 respectively, *n* = 79 and 52 for CTL and Il-1β respectively). **d** RT-qPCR of *xCT* mRNA normalized with the mean of 3 HKG mRNAs in isolated microglial cells cultured with or without IL-1β (50 ng/ml) (*n* = 4/group, Mann–Whitney). **e** Glutamate concentration (μM) measured in the supernatant of isolated microglial cells cultured with or without IL-1β (50 ng/ml) (*n* = 4/group, Mann–Whitney)

MPs produce large amounts of IL-1β, disturbing the ability of astrocytes (including Müller cells) to uptake glutamate; but they can also be a source of glutamate [36]. We thus next quantified the effect of IL-1β on microglial activation and glutamate release. Retinal explants were cultured for 18 h in the presence or not of IL-1β, and the phenotype of microglial cells was analyzed by immunochemistry. After 18 h of treatment with IL-1β, we observed a reduction in resident microglia cell perimeter and an increase in the surface of their soma, indicating microglial activation (Fig. 4a and b). mRNA expression of *xCT* in microglial cells isolated from retinal explants exposed to IL-1β was not found to be significantly modified compared with the unstimulated control (Fig. 4c). We next exposed isolated retinal microglial cells to IL-1β and quantified the extracellular glutamate concentration in the supernatant. Consistent with the absence of an *xCT* upregulation, IL-1β did not modify extracellular glutamate concentration (Fig. 4d) suggesting that microglial cells do not participate directly to the IL-1β-induced extracellular glutamate increase.

### IL-1β increases ionotropic glutamate receptor expression in the retina

**Fig. 5:**
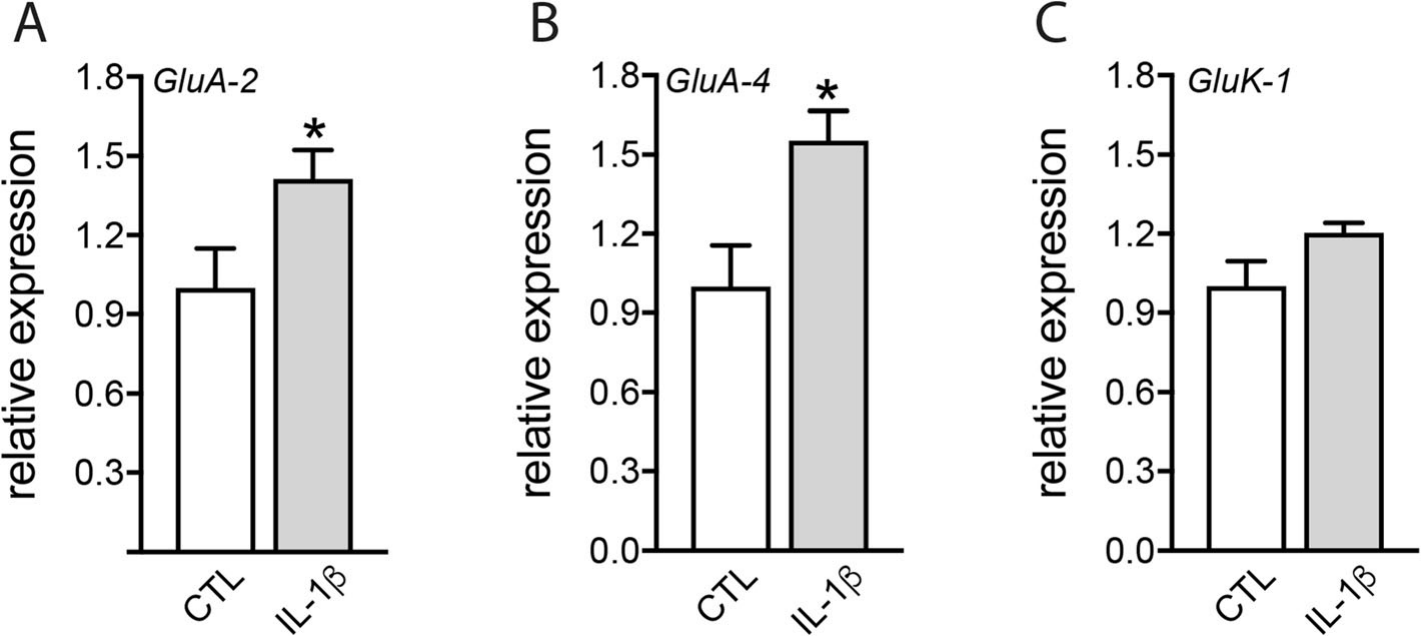
IL-1β increases ionotropic glutamate receptor expression in the retina. **a–c** RT-qPCR of *GluA-2*, *GluA-4*, and *GluK-1* mRNAs normalized with the mean of 3 HKG mRNAs in total mouse retinal explants after 18 h of culture with or without IL-1β (50 ng/ml) (*n* = 6/group, Mann–Whitney test, *p = 0.0350, *p = 0.0221, and *p* = 0.138 (NS) respectively)

Ionotropic glutamate receptors are localized on retinal neurons, including presynaptic rod and cone PRs [21–24]. Glutamate over-stimulation of these receptors leads to neuronal cell death in a process referred to excitotoxicity. RT-qPCR of mRNAs of glutamate receptor expression in retinal explants exposed or not to IL-1β (50 ng/ml) for 18 h revealed that IL-1β induced a 1.4- and 1.6-fold increase in mRNA levels of the AMPA glutamate receptor subunits *GluA-2* and *GluA-4* respectively (Fig. 5). These results suggest that IL-1β might increase the neuronal sensitivity to glutamate excitotoxicity, as it induces its ionotropic receptors in the retina.

### Glutamate excitotoxicity mediates monocyte and IL-1β-induced rod apoptosis

**Fig. 6:**
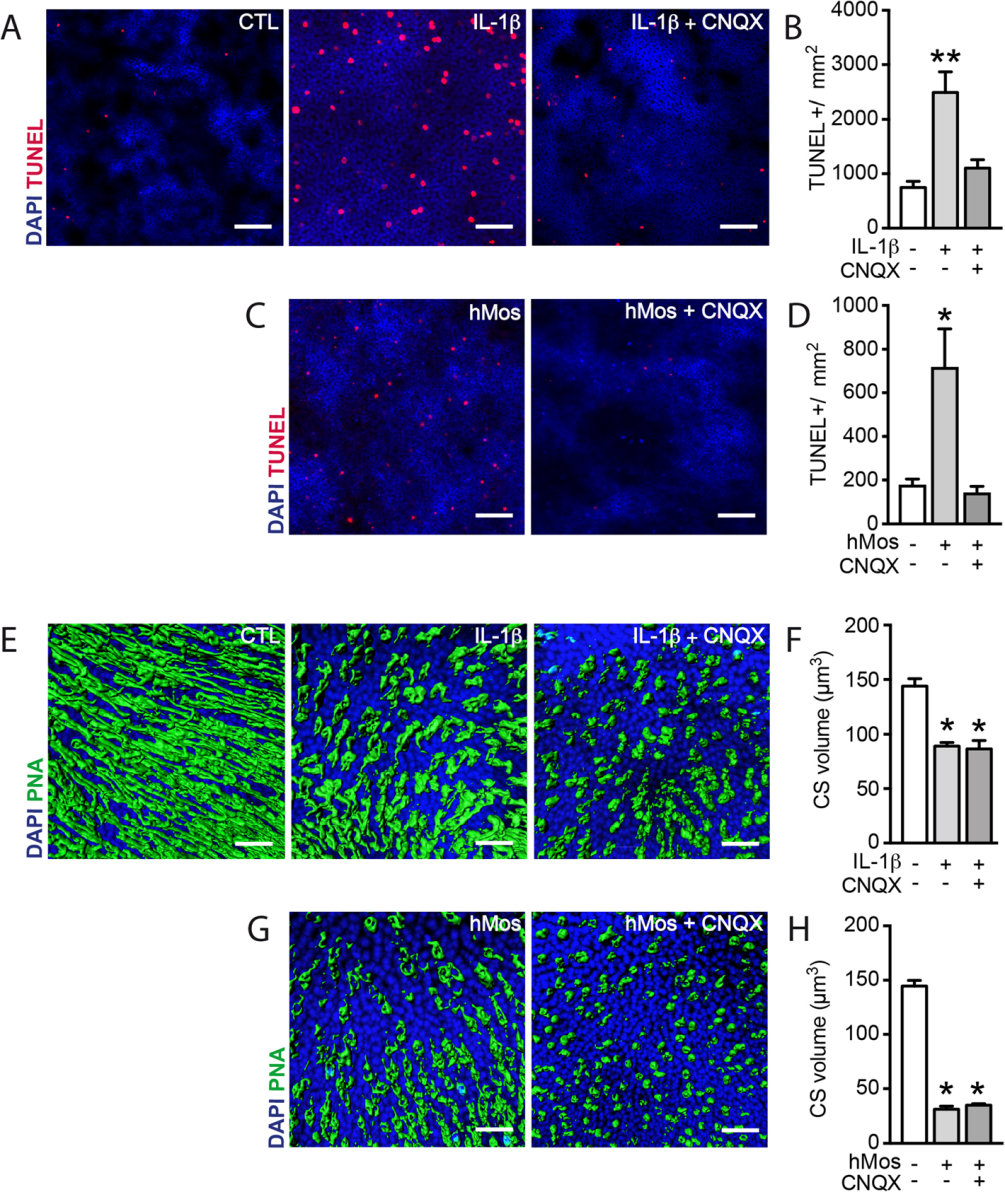
Glutamate excitotoxicity mediates monocyte and IL-1β-induced rod apoptosis. **a** Orthogonal projection of confocal Z stack images of the outer nuclear layer (ONL) of TUNEL (red)- and Hoechst nuclear dye (blue)-stained mouse retinal explants after 18 h of culture without IL-1β, with IL-1β (50 ng/ml) or with both IL-1β (50 ng/ml) and CNQX (100 μM). **b** Quantification of ONL TUNEL^+^ nuclei (*n* = 5/group, Kruskal–Wallis, Dunn’s post-test *p = 0.0029 versus “without IL-1β and CNQX”). **c** Orthogonal projection of confocal Z stack images of the ONL of TUNEL (red)- and Hoechst nuclear dye (blue)-stained mouse retinal explants after 18 h of culture without hMos, with hMos (100.000/explant), or with hMos and CNQX (100 μM). **d** Quantification of ONL TUNEL^+^ nuclei (*n* = 8/group, Kruskal–Wallis, Dunn’s post-test *p = 0.0294 versus “without hMos and CNQX”). **e** Oblique 3D reconstructions of confocal Z stack images of the PR segments of mouse retinal explants after 18 h of culture without IL-1β, with IL-1β (50 ng/ml), or with IL-1β and CNQX (100 μM) after peanut agglutinin (PNA, green) staining. **f** Quantification of PNA^+^ outer segment volume (μm^3^) (*n* = 4/group, Kruskal–Wallis, Dunn’s post-test *p = 0.0228, *p = 0.0279 respectively versus “without IL-1β and CNQX”). **g** Oblique 3D reconstructions of confocal Z stack images of the PR segments of mouse retinal explants after 18 h of culture without hMo, with hMo (100.000/explant), or with hMo and CNQX (100 μM) after peanut agglutinin (PNA, green) staining. **h** Quantification of PNA+ outer segment volume (μm^3^) (*n* = 3/group, Kruskal–Wallis, Dunn’s post-test *p = 0.0185 versus “without hMo and CNQX”)

**Fig. 7:**
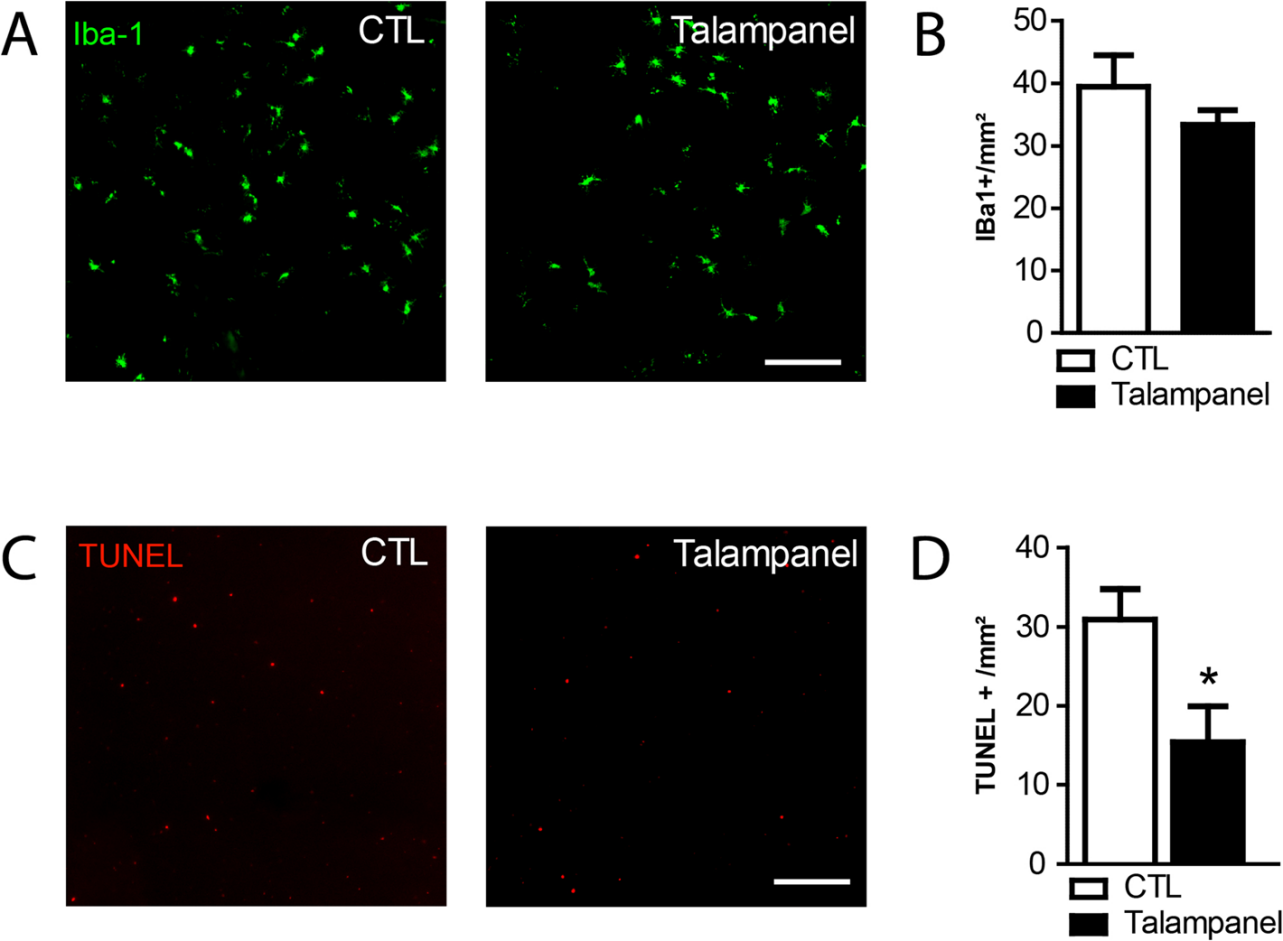
Talampanel prevents in vivo monocyte-induced photoreceptor apoptosis. Two- to three-month-old *Cx3cr1*^*GFP/GFP*^ mice were exposed for 4 days to constant light. On the fifth day, mice were injected IP every 2 h with Vehicle (CTL) or Talampanel in 0.5% tween-20. After 10 h, mice were sacrificed and Choiroid/RPE and retina were dissected. **a** Representative microphotographs of choroidal flatmounts of CTL- and Talampanel-treated mice immunostained with IBA1. **b** Quantification of the number of subretinal IBA1-positive cells in CTL- and Talampanel-treated mice. **c** Representative microphotographs of retinal flatmount of CTL- and Talampanel-treated mice stained by TUNEL. **d** Quantification of the number of TUNEL-positive cells in CTL- and Talampanel-treated mice. Scale bar 100 μm. (*n* = 8/group, Mann–Whitney test, *p = 0.021)

Our study shows that IL-1β does not directly induce PR apoptosis, but increases extracellular glutamate that is sufficient to induce rod apoptosis. We next tested if IL-1β rod-toxicity could be altered using CNQX (100 μM), a specific antagonist of AMPA/Kainate receptors that mediate glutamate exictotoxicity. TUNEL-stained retinal explants confirmed the robust increase of the number of TUNEL^+^ nuclei in the outer nuclear layer (ONL) after 18 h of IL-1β exposure (Fig. 6a). Quantifications of the number of these TUNEL^+^ nuclei demonstrated that the significant IL-1β-induced rod death in retinal explants could be completely prevented by the addition of CNQX (100 μM, Fig. 6b). The addition of MK-801, a specific antagonist of NMDA receptors, had no such effect (data not shown). Similarly, CNQX (100 μM) completely blunted the rod apoptosis induced by the co-culture of the retinal explants with hMos (Fig. 6c and d) that we previously showed to be due to IL-1β secreted by the hMos. In accordance with the lack of glutamate-induced CS degeneration (Fig. 1e), CNQX had no effect on IL-1β- (Fig. 6e and f) or hMo-induced (Fig. 6g and h) CS degeneration. To confirm these results in vivo, we next used the light-challenged *Cx3cr1*^*GFP/GFP*^ mice model. We showed in that model that primary accumulation of MPs leads to secondary IL-1β-dependent rod degeneration [12]. Mice were exposed for 4 days to constant light during which MPs accumulate in the subretinal space. The fifth day, mice were returned to normal light condition and treated or not with Talampanel a non-competitive inhibitor of AMPA receptors with neuroprotective effect against excitotoxicity [27]. Photoreceptor survival was evaluated after 10 h of treatment. Talampanel treatment did not modify the number of subretinal accumulated MPs (Fig. 7a, b) but reduced the number of TUNEL-positive photoreceptors (Fig. 7c, d). Taken together, our results indicate that IL-1β- and hMo-induced rod degenerations result from non-NMDA ionotropic receptor over-stimulation.

## Discussion

Mo-derived IL-1β induces rod PR cell death by an unknown mechanism during experimental subretinal inflammation and in diseases such as GA [12, 13]. We here show that PRs express little *Il1r1* compared with Müller cells and that exposure to IL-1β does not induce cell death in culture of isolated PRs but does so in retinal explants (Fig. 1). This suggests that IL-1β neurotoxic effect is indirect, as previously reported in other biological systems [26, 34, 37]. We here show that IL-1β induces an increase in extracellular glutamate and that glutamate alone is sufficient to induce rod PR apoptosis (Fig. 2), similar to IL-1β.

We first hypothesized that the IL-1β-induced increase of extracellular glutamate might result from a deficiency in glutamate reuptake [25]. However, we showed that the main glutamate transporter Eaat1/Glast transcription was unaffected. Similarly, we show that glutamate transporter *Eaat2* mRNA levels were only slightly reduced in retinal explants cultured with IL-1β and were not regulated in PRs (Fig. 3). In sharp contrast, we showed that IL-1β strongly induced the expression of xCT, an antiporter that imports cystine in exchange for exporting glutamate [33], in Müller cells (where it is most expressed)—but not in PRs (Fig. 3). Interestingly, Müller cells from IL-1β-treated retinal explants also displayed a reduced glutamine synthetase expression (Fig. 3). GS is an enzyme that catalyzes the transformation of glutamate in non-toxic glutamine, and its downregulation has previously been associated with Müller intracellular glutamate accumulation [38]. Accordingly, we measured an increased glutamate content in Müller glial cells when retinal explants were cultured with IL-1β for 18 h (Fig. 3). Taken together, our data show that IL-1β impairs retinal glutamate homeostasis on two levels: (i) IL-1β impairs the conversion of glutamate into glutamine in Müller cells, leading to increased intracellular glutamate concentrations, (ii) IL-1β induces increased xCT expression that likely increases glutamate export from Müller cells. Together this deregulation is likely responsible for the observed increase of extracellular glutamate induced by the exposure to IL-1β.

Glutamate exerts its excitotoxic effect on neurons via the over-stimulation of NMDA and non-NMDA (AMPA and Kainate) inotropic glutamate receptors [39, 40], which have been shown to be expressed in PR [21–24]. We here demonstrate in retinal explants that IL-1β increases AMPA receptor subunits *GluA-2* and *GluA-4* mRNAs, suggesting that IL-1β exposure could further sensitize retinal neurons to glutamate-induced excitotoxicity. Last but not least, using the AMPA/Kainate-receptor inhibitor CNQX, we demonstrate that the inhibition of non-NMDA receptors completely and efficiently prevented rod apoptosis in retinal explants cultured in the presence of IL-1β or Mo (Fig. 6). MK-801 inhibitor was unable to prevent rod PR apoptosis, indicating that rod PR degeneration is non-dependent on NMDA receptors. More importantly, we show that Talampanel a non-competitive inhibitor of AMPA receptor inhibited photoreceptor cell death in the light-challenged mice model of geographic atrophy (Fig. 7).

Interestingly, glutamate alone did not reproduce IL-1β-mediated cone segment shortening and CNQX did not prevent cone segment reduction induced by IL-1β or Mo. These results demonstrate that the mechanisms involved in cone segment shortening and rod PR degeneration are distinct. Indeed, cone segment maintenance depends on glucose uptake by cones [41]. The stimulation of glucose uptake in cones by rod-derived cone viability factor [41] or insulin [42] has been shown to rescue cones in a model of retinal degeneration.

We previously demonstrated the association of subretinal MP accumulation with rod degeneration in human samples of geographic atrophy [13]. Interestingly, the subretinal MP accumulated in a transitional zone, adjacent to the atrophy where a severe loss in rod numbers can be observed in the absence of RPE cell loss. We demonstrated that MP-derived IL-1β induces a similar rod degeneration in vitro and in vivo, which strongly suggests that subretinal MPs and MP-derived IL-1β participate in the degenerative changes in GA. However, the mechanism by which IL-1β induces rod death, in particular as these cells do not express the IL1R, remained unclear.

## Conclusion

Our manuscript demonstrates that IL-1β-dependent rod apoptosis is due to the deregulation of glutamate homeostasis and glutamate excitotoxicity. Our study emphasizes the importance of inflammation in the deregulation of glutamate homeostasis and provides a comprehensive mechanism of action for IL-1β-induced rod degeneration.

## Data Availability

All data generated or analyzed during this study are included in this published article. The datasets used and/or analyzed during the current study are also available from the corresponding author on reasonable request.

## Abbreviations

AMD: Age-related macular degeneration
CS: Cone outer segment
EAATs: Excitatory amino acid transporters
GA: Geographic atrophy
GS: Glutamine synthetase
HKG: Housekeeping genes
IL-1R1: Type I IL-1β receptor
MC: Microglial cell
Mo: Monocyte
MP: Mononuclear phagocyte
PR: Photoreceptor
RPE: Retinal pigment epithelium

## References

[1] Klein R, Peto T, Bird A, Vannewkirk MR (2004). The epidemiology of age-related macular degeneration. Am J Ophthalmol.

[2] Sarks JP, Sarks SH, Killingsworth MC (1988). Evolution of geographic atrophy of the retinal pigment epithelium. Eye (Lond).

[3] Chow A, Brown BD, Merad M (2011). Studying the mononuclear phagocyte system in the molecular age. Nat Rev Immunol.

[4] Gupta N, Brown KE, Milam AH (2003). Activated microglia in human retinitis pigmentosa, late-onset retinal degeneration, and age-related macular degeneration. Exp Eye Res.

[5] Guillonneau X, Eandi CM, Paques M, Sahel JA, Sapieha P, Sennlaub F (2017). On phagocytes and macular degeneration. Prog Retin Eye Res.

[6] Sennlaub F, Auvynet C, Calippe B, Lavalette S, Poupel L, Hu SJ, Dominguez E, Camelo S, Levy O, Guyon E (2013). CCR2(+) monocytes infiltrate atrophic lesions in age-related macular disease and mediate photoreceptor degeneration in experimental subretinal inflammation in Cx3cr1 deficient mice. EMBO Mol Med.

[7] Guo C, Otani A, Oishi A, Kojima H, Makiyama Y, Nakagawa S, Yoshimura N (2012). Knockout of ccr2 alleviates photoreceptor cell death in a model of retinitis pigmentosa. Exp Eye Res.

[8] Rutar M, Natoli R, Provis JM (2012). Small interfering RNA-mediated suppression of Ccl2 in Muller cells attenuates microglial recruitment and photoreceptor death following retinal degeneration. J Neuroinflammation.

[9] Cruz-Guilloty F, Saeed AM, Echegaray JJ, Duffort S, Ballmick A, Tan Y, Betancourt M, Viteri E, Ramkhellawan GC, Ewald E (2013). Infiltration of proinflammatory m1 macrophages into the outer retina precedes damage in a mouse model of age-related macular degeneration. Int J Inflam.

[10] Kohno H, Chen Y, Kevany BM, Pearlman E, Miyagi M, Maeda T, Palczewski K, Maeda A (2013). Photoreceptor proteins initiate microglial activation via toll-like receptor 4 in retinal degeneration mediated by all-trans-retinal. J Biol Chem.

[11] Shaftel SS, Griffin WS, O’Banion MK (2008). The role of interleukin-1 in neuroinflammation and Alzheimer disease: an evolving perspective. J Neuroinflammation.

[12] Hu SJ, Calippe B, Lavalette S, Roubeix C, Montassar F, Housset M, Levy O, Delarasse C, Paques M, Sahel JA (2015). Upregulation of P2RX7 in Cx3cr1-deficient mononuclear phagocytes leads to increased interleukin-1beta secretion and photoreceptor neurodegeneration. J Neurosci.

[13] Eandi CM, Charles Messance H, Augustin S, Dominguez E, Lavalette S, Forster V, Hu SJ, Siquieros L, Craft CM, Sahel JA, et al. Subretinal mononuclear phagocytes induce cone segment loss via IL-1beta. Elife. 2016;5. 10.7554/eLife.16490 PMC4969036 27438413

[14] Huang Y, Smith DE, Ibanez-Sandoval O, Sims JE, Friedman WJ (2011). Neuron-specific effects of interleukin-1beta are mediated by a novel isoform of the IL-1 receptor accessory protein. J Neurosci.

[15] Fogal B, Hewett JA, Hewett SJ (2005). Interleukin-1β potentiates neuronal injury in a variety of injury models involving energy deprivation. J Neuroimmunol.

[16] Scuderi S, D’Amico AG, Federico C, Saccone S, Magro G, Bucolo C, Drago F, D’Agata V (2015). Different retinal expression patterns of IL-1alpha, IL-1beta, and their receptors in a rat model of type 1 STZ-induced diabetes. J Mol Neurosci.

[17] Harada T, Harada C, Kohsaka S, Wada E, Yoshida K, Ohno S, Mamada H, Tanaka K, Parada LF, Wada K (2002). Microglia-Muller glia cell interactions control neurotrophic factor production during light-induced retinal degeneration. J Neurosci.

[18] Natoli R, Rutar M, Lu YZ, Chu-Tan JA, Chen Y, Saxena K, Madigan M, Valter K, Provis JM (2016). The role of pyruvate in protecting 661 W photoreceptor-like cells against light-induced cell death. Curr Eye Res.

[19] Donovan M, Carmody RJ, Cotter TG (2001). Light-induced photoreceptor apoptosis in vivo requires neuronal nitric-oxide synthase and guanylate cyclase activity and is caspase-3-independent. J Biol Chem.

[20] Delyfer MN, Forster V, Neveux N, Picaud S, Leveillard T, Sahel JA (2005). Evidence for glutamate-mediated excitotoxic mechanisms during photoreceptor degeneration in the rd1 mouse retina. Mol Vis.

[21] Fletcher EL, Hack I, Brandstatter JH, Wassle H (2000). Synaptic localization of NMDA receptor subunits in the rat retina. J Comp Neurol.

[22] Harvey DM, Calkins DJ (2002). Localization of kainate receptors to the presynaptic active zone of the rod photoreceptor in primate retina. Vis Neurosci.

[23] Haumann I, Junghans D, Anstotz M, Frotscher M (2017). Presynaptic localization of GluK5 in rod photoreceptors suggests a novel function of high affinity glutamate receptors in the mammalian retina. PLoS One.

[24] Lin Y, Jones BW, Liu A, Vazquez-Chona FR, Lauritzen JS, Ferrell WD, Marc RE (2012). Rapid glutamate receptor 2 trafficking during retinal degeneration. Mol Neurodegener.

[25] Chen C, Chen H, Xu C, Zhong Y, Shen X (2014). Role of interleukin-1beta in hypoxia-induced depression of glutamate uptake in retinal Muller cells. Graefes Arch Clin Exp Ophthalmol.

[26] Fogal B, Li J, Lobner D, McCullough LD, Hewett SJ: System x(c)- activity and astrocytes are necessary for interleukin-1 beta-mediated hypoxic neuronal injury. J Neurosci, vol. 27. pp. 10094-10105: Society for Neuroscience; 2007:10094-10105. 10.1523/JNEUROSCI.2459-07.2007 PMC6672668 17881516

[27] Erdo F, Berzsenyi P, Nemet L, Andrasi F (2006). Talampanel improves the functional deficit after transient focal cerebral ischemia in rats. A 30-day follow up study. Brain Res Bull.

[28] Grosche A, Hauser A, Lepper MF, Mayo R, von Toerne C, Merl-Pham J, Hauck SM (2016). The proteome of native adult muller glial cells from murine retina. Mol Cell Proteomics.

[29] Vallazza-Deschamps G, Cia D, Gong J, Jellali A, Duboc A, Forster V, Sahel JA, Tessier LH, Picaud S (2005). Excessive activation of cyclic nucleotide-gated channels contributes to neuronal degeneration of photoreceptors. Eur J Neurosci.

[30] Ye L, Huang Y, Zhao L, Li Y, Sun L, Zhou Y, Qian G, Zheng JC (2013). IL-1beta and TNF-alpha induce neurotoxicity through glutamate production: a potential role for neuronal glutaminase. J Neurochem.

[31] Prow NA, Irani DN (2008). The inflammatory cytokine, interleukin-1 beta, mediates loss of astroglial glutamate transport and drives excitotoxic motor neuron injury in the spinal cord during acute viral encephalomyelitis. J Neurochem.

[32] Niklaus Stephanie, Cadetti Lucia, vom Berg-Maurer Colette M., Lehnherr André, Hotz Adriana L., Forster Ian C., Gesemann Matthias, Neuhauss Stephan C.F. (2017). Shaping of Signal Transmission at the Photoreceptor Synapse by EAAT2 Glutamate Transporters. eneuro.

[33] Lewerenz Jan, Hewett Sandra J., Huang Ying, Lambros Maria, Gout Peter W., Kalivas Peter W., Massie Ann, Smolders Ilse, Methner Axel, Pergande Mathias, Smith Sylvia B., Ganapathy Vadivel, Maher Pamela (2013). The Cystine/Glutamate Antiporter System xc− in Health and Disease: From Molecular Mechanisms to Novel Therapeutic Opportunities. Antioxidants & Redox Signaling.

[34] Jackman NA, Uliasz TF, Hewett JA, Hewett SJ (2010). Regulation of system x_c_− activity and expression in astrocytes by interleukin-1β. Glia.

[35] Shi J, He Y, Hewett SJ, Hewett JA (2016). Interleukin 1beta regulation of the system xc- substrate-specific subunit, xCT, in primary mouse astrocytes involves the RNA-binding protein HuR. J Biol Chem.

[36] Kigerl KA, Ankeny DP, Garg SK, Wei P, Guan Z, Lai W, McTigue DM, Banerjee R, Popovich PG (2012). System x(c)(-) regulates microglia and macrophage glutamate excitotoxicity in vivo. Exp Neurol.

[37] Koprich JB, Reske-Nielsen C, Mithal P, Isacson O (2008). Neuroinflammation mediated by IL-1beta increases susceptibility of dopamine neurons to degeneration in an animal model of Parkinson’s disease. J Neuroinflammation.

[38] Barnett NL, Pow DV, Robinson SR (2000). Inhibition of Muller cell glutamine synthetase rapidly impairs the retinal response to light. Glia.

[39] Choi DW, Maulucci-Gedde M, Kriegstein AR (1987). Glutamate neurotoxicity in cortical cell culture. J Neurosci.

[40] Liu SJ, Zukin RS (2007). Ca2+-permeable AMPA receptors in synaptic plasticity and neuronal death. Trends Neurosci.

[41] Ait-Ali N, Fridlich R, Millet-Puel G, Clerin E, Delalande F, Jaillard C, Blond F, Perrocheau L, Reichman S, Byrne LC (2015). Rod-derived cone viability factor promotes cone survival by stimulating aerobic glycolysis. Cell.

[42] Punzo C, Kornacker K, Cepko CL (2009). Stimulation of the insulin/mTOR pathway delays cone death in a mouse model of retinitis pigmentosa. Nat Neurosci.

